# Evidence that KARRIKIN-INSENSITIVE2 (KAI2) Receptors may Perceive an Unknown Signal that is not Karrikin or Strigolactone

**DOI:** 10.3389/fpls.2015.01219

**Published:** 2016-01-08

**Authors:** Caitlin E. Conn, David C. Nelson

**Affiliations:** Department of Genetics, University of Georgia, AthensGA, USA

**Keywords:** karrikins, strigolactones, signaling, evolution, hormones

## Abstract

The α/β-hydrolases KAI2 and D14 are paralogous receptors for karrikins and strigolactones, two classes of plant growth regulators with butenolide moieties. KAI2 and D14 act in parallel signaling pathways that share a requirement for the F-box protein MAX2, but produce distinct growth responses by regulating different members of the SMAX1-LIKE/D53 family. *kai2* and *max2* mutants share seed germination, seedling growth, leaf shape, and petiole orientation phenotypes that are not found in *d14* or SL-deficient mutants. This implies that KAI2 recognizes an unknown, endogenous signal, herein termed KAI2 ligand (KL). Recent studies of ligand-specificity among *KAI2* paralogs in basal land plants and root parasitic plants suggest that karrikin and strigolactone perception may be evolutionary adaptations of KL receptors. Here we demonstrate that evolutionarily conserved *KAI2c* genes from two parasite species rescue multiple phenotypes of the *Arabidopsis kai2* mutant, unlike karrikin-, and strigolactone-specific *KAI2* paralogs. We hypothesize that KAI2c proteins recognize KL, which could be an undiscovered hormone.

Strigolactones (SLs) are a family of plant hormones that control shoot branching, root architecture, secondary growth, and senescence (Gomez-Roldan et al., 2008; Umehara et al., 2008; Agusti et al., 2011; Kapulnik et al., 2011; Rasmussen et al., 2012; Yamada et al., 2014; Ueda and Kusaba, 2015). SLs are also exuded from plant roots into the rhizosphere, particularly under low nutrient conditions, where they promote symbiotic associations with arbuscular mycorrhizal fungi (Akiyama et al., 2005). This fungal recruitment mechanism is exploited by root parasitic plants in the Orobanchaceae family, which use SLs as a way to detect a nearby host and germinate (Yoneyama et al., 2010). SLs are produced from carotenoids via a carlactone intermediate (Alder et al., 2012; Al-Babili and Bouwmeester, 2015). The basic structure of SLs consists of a tricyclic lactone (ABC ring) connected by an enol-ether bond to a butenolide moiety (D ring). The D ring is oriented in the 2′*R* configuration in all strigolactones that have been identified from plants (Xie et al., 2013; Zwanenburg and Pospisil, 2013).

KARs and SLs are not interchangeable signals. KARs do not rescue the excess branching phenotype of SL-deficient mutants (Nelson et al., 2011), and SLs and carlactone do not promote germination of *Arabidopsis* seed (Scaffidi et al., 2013, 2014). Some confusion about SL roles has arisen due to the widespread use of a synthetic strigolactone racemic mixture, *rac*-GR24. It has only recently been recognized that the two enantiomers that comprise *rac*-GR24 have different effects due to opposite orientations of the D ring: GR24^5DS^ mimics the configuration and activity of the natural strigolactone 5-deoxystrigol (5DS), but GR24*^ent^*^-5DS^ has an unnatural 2′*S* configuration and primarily acts through the KAR signaling pathway (Scaffidi et al., 2014).

## Mechanisms of KARRIKIN and STRIGOLACTONE Perception

Although KAR and SL have unique sources and effects on plant development, genetic studies have shown that both signals act through similar mechanisms. Growth responses to both KAR and SL require the F-box protein MAX2 (Nelson et al., 2011). Specific responses to karrikins and strigolactones respectively require the α/β-hydrolases KARRIKIN INSENSITIVE2 (KAI2) and DWARF14 (D14), which are ancient paralogs. *D14* is only present in vascular plants, where it likely arose through duplication of *KAI2* (Delaux et al., 2012; Waters et al., 2012). In *Arabidopsis, kai2/htl* mutants have increased seed dormancy, elongated hypocotyls, smaller cotyledons, hyponastic petioles, and altered leaf morphology, but have normal shoot branching (Sun and Ni, 2011; Waters et al., 2012). *d14* mutants in rice, petunia, and *Arabidopsis* have increased axillary branching (or tillering), reduced stature, and delayed senescence; these phenotypes are shared with SL-deficient mutants, but are not rescued by SL application (Arite et al., 2009; Hamiaux et al., 2012; Waters et al., 2012; Yamada et al., 2014; Ueda and Kusaba, 2015). Unlike *kai2*, however, *d14* and SL-deficient mutants in *Arabidopsis* have wildtype germination and seedling growth (Nelson et al., 2011; Shen et al., 2012; Waters et al., 2012). *max2* phenotypes are a complex union of *kai2* and *d14* phenotypes (Waters et al., 2012; Soundappan et al., 2015).

Substantial evidence supports the hypothesis that KAI2 and D14 are receptors for KAR and SL, respectively. Several types of *in vitro* assays have shown that KAI2 binds KAR_1_, and that D14 binds and hydrolyzes GR24 (Hamiaux et al., 2012; Guo et al., 2013; Kagiyama et al., 2013; Nakamura et al., 2013; Zhao et al., 2013, 2015). Protein-ligand complexes of KAI2-KAR_1_ and D14 with GR24 and its hydrolytic products have been resolved by X-ray crystallography (Guo et al., 2013; Nakamura et al., 2013; Zhao et al., 2013, 2015). D14 function requires an active catalytic site, but the byproducts of GR24 hydrolysis do not rescue the *d14/dad2* mutant in petunia (Hamiaux et al., 2012). This indicates that signal transduction is achieved by the act of ligand binding or hydrolysis. Indeed, SL promotes the association of D14 with MAX2/D3 and DWARF53 (D53) in rice, which causes polyubiquitination and proteolysis of D53 (Jiang et al., 2013; Zhou et al., 2013, Zhao et al., 2014). Degradation of D53 is necessary for SL signal transduction (Jiang et al., 2013; Zhou et al., 2013). D14 does not show obvious conformational changes during SL binding or hydrolysis, but in the presence of GR24 it undergoes thermal destabilization that is enhanced by MAX2/D3 interactions (Hamiaux et al., 2012; Zhao et al., 2015). D14 interactions with MAX2/D3 and D53 are selectively activated by 2′*R* SL stereoisomers (Umehara et al., 2015; Zhao et al., 2015). *Arabidopsis* D14 destabilizes in the presence of either GR24^5DS^ or GR24*^ent-^*^5DS^, but it is primarily responsive to GR24^5DS^ *in vivo* (Scaffidi et al., 2014; Waters et al., 2015b). In *Arabidopsis*, the D53 orthologs SMXL6, SMXL7, and SMXL8 mediate growth responses to SL downstream of MAX2, interact with D14, and are also targeted for degradation in a D14-dependent manner following GR24 treatment (Soundappan et al., 2015; Umehara et al., 2015; Wang et al., 2015). KAR perception by KAI2 is hypothesized to trigger similar degradation of SMAX1, a D53/SMXL6/7/8 paralog in *Arabidopsis* that controls seed germination, seedling growth, and leaf development (Stanga et al., 2013; Bennett and Leyser, 2014; Soundappan et al., 2015).

## *kai2* Phenotypes Suggest Insensitivity to an Endogenous, Non-Strigolactone Ligand

In addition to KAR-insensitivity, *kai2* and *max2* mutants share phenotypes of increased seed dormancy and reduced photomorphogenic growth in seedlings. These phenotypes are opposite to the effects of KAR treatment, and are not observed in *d14* or SL-deficient mutants (Nelson et al., 2011; Shen et al., 2012). This observation provided the first evidence that *KAI2* and *MAX2* are required for responses to an endogenous, non-SL signal (Flematti et al., 2013; Waters et al., 2014). In addition, *Arabidopsis* KAI2 hydrolyzes GR24*^ent^*^-5DS^ and undergoes thermal destabilization in its presence, but KAI2 is not active toward GR24^5DS^ (Waters et al., 2015b). These data support observations that GR24*^ent^*^-5DS^, but not GR24^5DS^ or carlactone, enhances *Arabidopsis* germination, which is controlled by KAI2 but not D14 (Scaffidi et al., 2013, 2014). Thus *Arabidopsis* KAI2 has the capacity to respond to more than one type of non-SL butenolide compound, but is not involved in SL signaling. We propose that KARs and GR24*^ent^*^-5DS^ can fortuitously substitute for its endogenous signal(s), which we herein refer to as KAI2 ligand (KL). Like strigolactones, KL is not necessarily a single compound and may be a class of similar molecules.

An alternative explanation for *kai2* mutant phenotypes is that KAI2 is always active, but KAR enhances its activity. For example, KAI2 could have a weak physical interaction with a signaling partner that is strengthened by KAR. In this case *kai2* phenotypes could result from a loss of constitutive, ligand-independent KAI2 activity rather than a lack of KL perception. Two observations counter this idea. First, a Ser-His-Asp catalytic triad is conserved throughout KAI2 proteins found in land plants, indicating selective pressure to retain catalytic activity. Indeed the catalytic Ser is required for KAI2 hydrolytic activity and rescue of *kai2* mutants (Waters et al., 2014, 2015a,b). Thus hydrolysis-independent activity is unlikely to have functional significance. Second, overexpression of *KAI2* does not induce constitutive KAR response phenotypes in seedlings, as would be expected if basal activity were increased (Waters and Smith, 2013). However, it does cause hypersensitivity to KAR treatment, which is consistent with an increased capacity for ligand-activated signaling. It must be noted that KAI2 overexpression does reduce seed dormancy (Waters and Smith, 2013); we propose that this observation reflects that KL is a limiting factor for KAI2 activity in seedlings but not in seed.

## A Lycophyte Kai2 may be Kl-Specific

Further support for the existence of KL comes from the evolutionary history of KAR and SL signaling. *KAI2* homologs are found in charophyte green algae and the basal land plants *Physcomitrella patens* and *Marchantia polymorpha* (Delaux et al., 2012; Waters et al., 2015b). *D14* orthologs are found only in vascular plants, and most likely arose from a duplication of *KAI2* (Delaux et al., 2012; Waters et al., 2012). This indicates that KAI2 signaling preceded strigolactone perception by D14, but does not clarify the ligand-specificities of basal KAI2. Indeed strigolactones have been identified in basal land plants, and *rac*-GR24 can induce growth responses in bryophytes and Charales (Proust et al., 2011; Delaux et al., 2012; Hoffmann et al., 2014). Waters et al. (2015b) recently investigated the biochemical activities and functions of *KAI2* homologs in *S. moellendorfii* and *M. polymorpha*. SmKAI2b, one of two *KAI2* paralogs in *S. moellendorfii*, has hydrolytic activity against GR24^5DS^ that suggests it might be a SL receptor, but it does not rescue *Arabidopsis kai2* or *d14* mutants. In contrast, *SmKAI2a* partially rescues *kai2* seedling growth and fully rescues *kai2* leaf morphology. The ability of *SmKAI2a* to rescue *Arabidopsis kai2* is dependent upon an active catalytic site. Interestingly, *SmKAI2a* does not confer responses to KARs, GR24, or carlactone in transgenic seedling growth assays. Although SmKAI2a has *in vitro* hydrolase activity against a generic substrate, it does not hydrolyze either 2′*R* or 2′*S* GR24 stereoisomers. And unlike AtKAI2 and AtD14, it does not undergo thermal stability shifts in the presence of either GR24 stereoisomer (Waters et al., 2015b). As *SmKAI2a* partially rescues *kai2* – despite having no apparent KAR or SL receptor activity – it likely recognizes endogenous substrates that are similar or the same in *Selaginella* and *Arabidopsis*, which we propose to be KL.

## Kai2 Paralogs in Parasitic Plants have Diversified Ligand-Specificities

**FIGURE 1:**
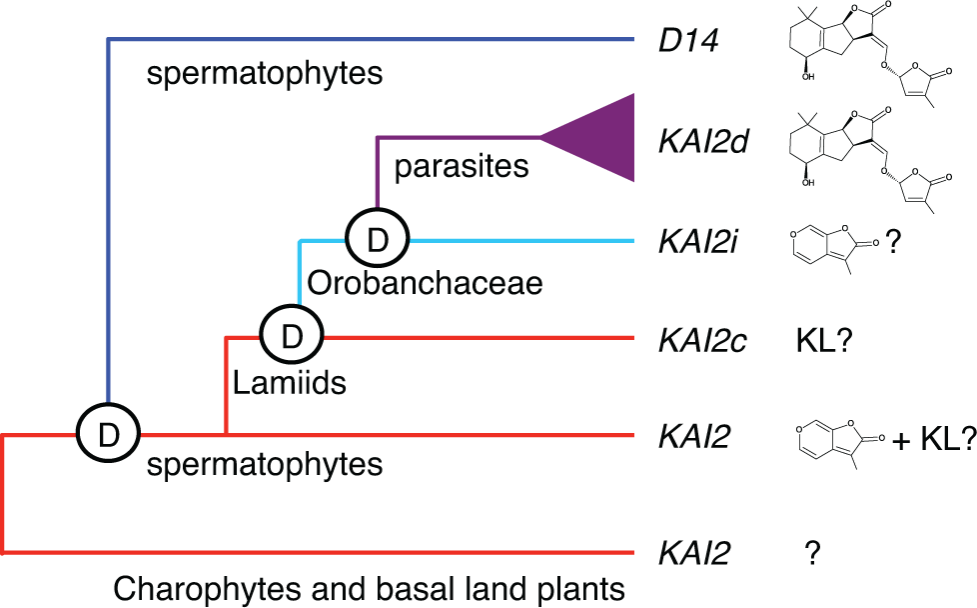
**Model of *KAI2* and *D14* evolution.** *KAI2* orthologs are present in charophyte algae and basal land plants. *D14* likely arose by duplication of *KAI2* prior to the evolution of spermatophytes. Although the timing of this duplication is uncertain, a potential *D14* ortholog is present in *Selaginella moellendorfii*. We hypothesize that most spermatophyte KAI2 are KL and/or KAR receptors. In parasitic plants, *KAI2* duplication and evolution have produced three clades. KAI2c is hypothesized to be KL-specific, KAI2i may be KAR-specific, and KAI2d are SL-responsive. Reprinted from Conn et al. (2015) with permission from AAAS.

We also discovered evidence for KL-specificity among evolutionarily conserved *KAI2* paralogs in root parasitic plants (Conn et al., 2015). A broad range of parasitism is found within the Orobanchaceae family, from a single non-parasitic genus, to facultative parasites that do not require a host, to obligate parasites that are dependent upon a host. The obligate parasite taxa include several highly destructive agricultural weeds, such as witchweed (*Striga* spp.) and broomrapes (*Orobanche, Phelipanche* spp.), which germinate in response to SLs but not KARs. As SLs indicate the presence of a host, and obligate parasites only survive if they attach to a host root within days of germination, this response is a critical adaptation. We found that *KAI2* has undergone extensive gene duplication and diversification of ligand specificity in parasite genomes. *KAI2* genes in the Lamiales order are grouped into three clades: conserved (*KAI2c*), intermediate (*KAI2i*), and divergent (*KAI2d*). *KAI2c* paralogs are the most broadly distributed in Lamiales species. The *KAI2c* clade is under the strongest purifying selection and has the highest similarity to *KAI2* genes in non-parasitic angiosperms. *KAI2i* paralogs are under intermediate purifying selection, and are found in most Lamiales genomes. *KAI2d* paralogs comprise a parasite-specific clade that has undergone significant expansion and has evolved the most quickly. Homology modeling predicts a high degree of similarity between the ligand-binding pockets of KAI2c paralogs and AtKAI2, whereas KAI2d pockets are significantly enlarged and have diverse shapes. Our current evolutionary model is that *KAI2c* and *KAI2i* arose through *KAI2* gene duplication in the Lamiids, and *KAI2d* arose through further duplication and neofunctionalization in the parasitic Orobanchaceae lineage (Conn et al., 2015) (**Figure 1**).

We investigated the ligand-specificity of *KAI2* genes from *Striga hermonthica* and *Phelipanche aegyptiaca* by cross-species complementation of *Arabidopsis kai2*. *KAI2* transgenes from the three clades confer different germination phenotypes: (1) *KAI2c* from *P. aegyptiaca* (*PaKAI2c*) rescues the *Arabidopsis kai2* seed dormancy phenotype, but does not confer responses to KARs or *rac*-GR24; (2) *KAI2i* from *S*. *hermonthica* (*ShKAI2i*) does not rescue *kai2* dormancy, but confers germination responses to KARs; and (3) three *KAI2d* paralogs do not rescue *kai2* dormancy, but confer germination responses to *rac*-GR24. These *KAI2d* confer stronger germination responses to GR24 stereoisomers with SL-like 2′*R* configurations than those with unnatural 2′*S* configurations. Thus, KAI2d in parasitic weeds are likely to be SL receptors that function in host-responsive seed germination (Conn et al., 2015). An independent cross-species complementation analysis of *KAI2*/*HTL* paralogs from *Striga hermonthica* is consistent with our findings. The *KAI2i* class paralogs *ShHTL2* and *ShHTL3* confer KAR-specific germination to *Arabidopsis kai2*, and six *KAI2d* class paralogs (*ShHTL4*-*ShHTL9*) have SL-specific functions (Toh et al., 2015).

The conclusions from these functional approaches are supported by *in vitro* studies of ligand selectivity among *KAI2/HTL* paralogs in *S. hermonthica* (Tsuchiya et al., 2015). The KAI2c paralog (ShHTL1) does not hydrolyze *rac*-GR24, but eight KAI2d paralogs (ShHTL4 to ShHTL11) hydrolyze *rac*-GR24 and show a range of affinities for different strigolactones. KAI2i paralogs (ShHTL2 and ShHTL3) have weaker hydrolytic activities on *rac*-GR24 and lower *in vitro* affinities for strigolactones than any of the KAI2d paralogs (Tsuchiya et al., 2015).

*PaKAI2c* rescues *Arabidopsis kai2* seed dormancy but does not confer KAR or GR24 responses, which suggests that this protein is specific for KL. *ShKAI2c* does not appear to be active in *Arabidopsis* seed; like *SmKAI2a*, however, it could encode a KL receptor that has reduced function in *Arabidopsis* compared to its native context. Reduced function could be due to inefficient interactions with *Arabidopsis* signaling partners, which is an inherent limitation of the cross-species complementation approach. Functional rescue at later stages of growth, as seen for *SmKAI2a*, could result from a lower signaling threshold, or changes in KAI2 signaling partner availability. Therefore we investigated whether *PaKAI2c* and *ShKAI2c* can rescue *Arabidopsis kai2* phenotypes after germination.

## New Evidence that Parasite Kai2c may be Kl-Specific Receptors

**FIGURE 2:**
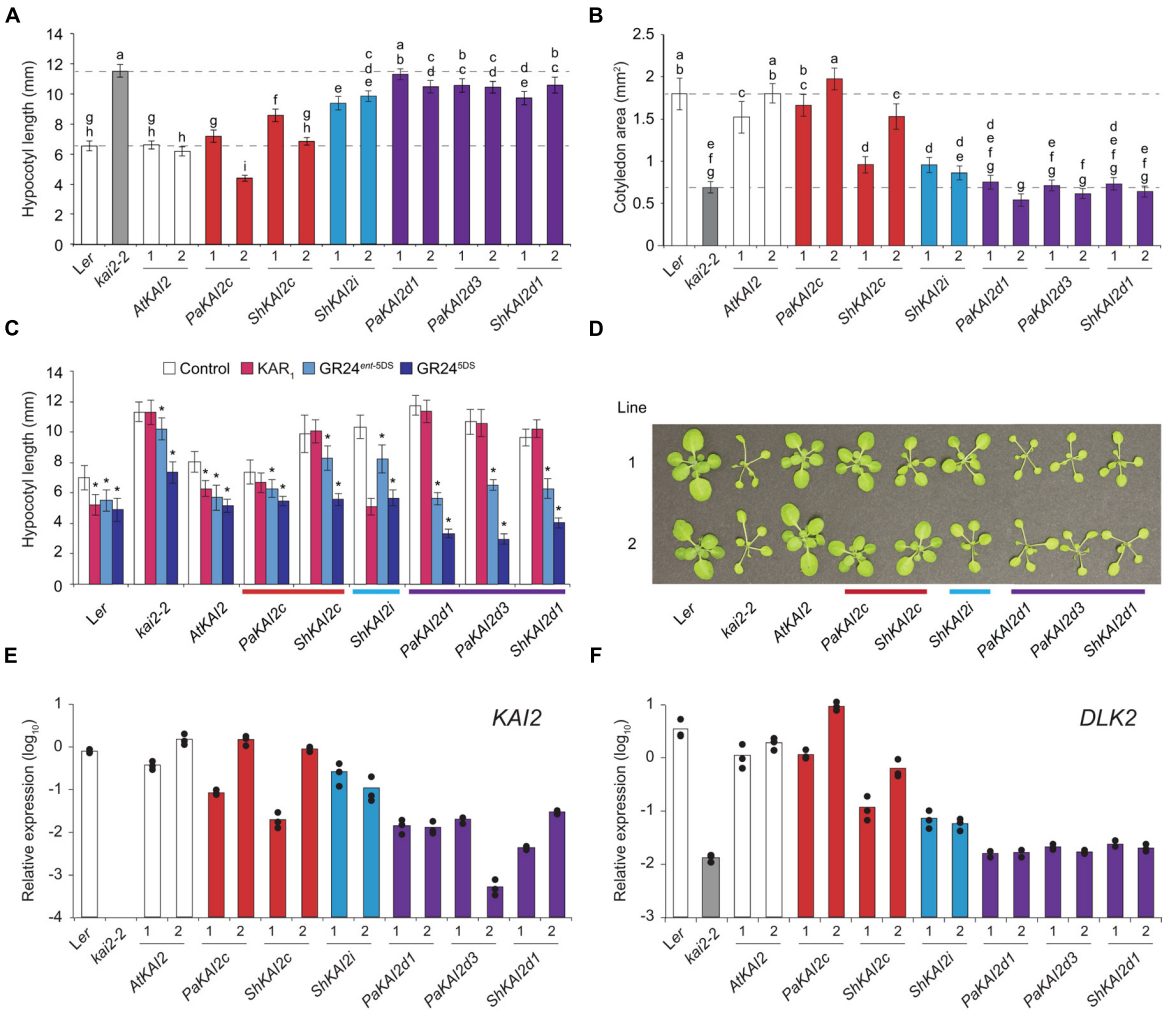
***KAI2* from parasites confer different growth responses to *Arabidopsis kai2*. (A)** Hypocotyl length and **(B)** cotyledon area of 10 day-old seedlings grown under short-day conditions. *KAI2* transgenes from *Arabidopsis thaliana* (At), *Striga hermonthica* (Sh), and *Phelipanche aegyptiaca* (Pa) under control of an *AtKAI2* promoter are in the *Arabidopsis kai2-2* mutant background. Data are shown for two homozygous, independent transgenic lines per transgene. Mean ±99% CI, *n* = 45. Tukey–Kramer HSD test, *p* < 0.01. **(C)** Hypocotyl length of 10 day-old seedlings grown on 1 μM KAR_1_, GR24*^ent-^*^5DS^, and GR24^5DS^ as described above. Transgenic line 1 was tested. Mean ±99% CI, *n* = 15. Dunnett’s test comparing treatments to control, ^∗^*p* < 0.01. **(D)** 31 day-old rosettes grown under short-day conditions (white light, 65–136 μE). A representative plant from each of two independent transgenic lines is shown per transgene. **(E)** Expression of *KAI2* and **(F)** *DLK2* in 10 day-old seedlings, relative to the *CACS* reference gene. In **(E)**, *KAI2* expression is of wildtype *KAI2* (L*er*) or the respective parasite *KAI2* transgene (all other lines). Columns represent the mean expression of three biological replicates (at least two technical replicates each), each of which is indicated by a black dot.

*PaKAI2c* completely restored, and *ShKAI2c* mostly restored, *kai2* hypocotyl length and cotyledon size to wildtype (**Figures 2A,B**). *ShKAI2i*, which confers KAR-specific germination responses, partially rescued *kai2* hypocotyl length but had little effect on cotyledon size. In comparison, the strigolactone-responsive genes *PaKAI2d1, PaKAI2d3*, and *ShKAI2d1* had little or no effect on either aspect of *kai2* seedling growth (**Figures 2A,B**). We tested the hypocotyl elongation responses of these transgenic lines to KAR_1_ and two GR24 stereoisomers (**Figure 2C**). All transgenic lines were expected to be GR24 responsive due to D14 activity (Scaffidi et al., 2014). *PaKAI2c* and *ShKAI2c* did not confer responses to KAR_1_ and were not more sensitive to GR24^5DS^ than *kai2*. *ShKAI2i* seedlings had a strong response to KAR_1_, consistent with this transgene’s KAR-specific germination responses (Conn et al., 2015). *PaKAI2d1, PaKAI2d3*, and *ShKAI2d1* seedlings were insensitive to KAR_1_ and more responsive to GR24*^ent-^*^5DS^ and GR24^5DS^ than *kai2*. Only *ShKAI2d1* confers germination responses to GR24*^ent^*^-5DS^, but all three *KAI2d* lines responded to it at the seedling stage. Finally, we examined rosette phenotypes (**Figure 2D**). *PaKAI2c* and to a lesser extent *ShKAI2c* rescued *kai2* leaf morphology, but *KAI2d* transgenes did not. As in seedlings, *ShKAI2i* partially rescued the *kai2* leaf phenotype.

Thus *PaKAI2c* rescues all visible *kai2* mutant phenotypes, and *ShKAI2c* – like *SmKAI2a* – can partially rescue *kai2* at certain stages of development. These results suggest that receptors in the *KAI2c* clade in parasites recognize KL. To a lesser extent *ShKAI2i*, which has a stronger response to KAR_1_ than any other treatments, was able to partially rescue *kai2*. This may reflect a residual affinity for KL in these KAR-responsive receptors. However, the SL-specific *KAI2d* had little effect on *kai2* phenotypes, even though they can function in an *Arabidopsis* context and confer sensitive seed and seedling responses to GR24. Toh et al. (2015) also found that strigolactone-specific *KAI2d* transgenes from *Striga hermonthica* do not rescue *Arabidopsis kai2* seed dormancy.

The *PaKAI2c* and *ShKAI2c* transgenic lines rescued *kai2* mutant phenotypes to different extents (**Figures 2A,B**). We investigated whether transgene expression level contributed to these differences. We found a correlation between transgene expression, rescue, and expression of the KAR/SL reporter gene *DLK2* (**Figure 2**). Interestingly, *KAI2d* transgenes were expressed at a much lower level than *KAI2c* and *KAI2i* (**Figure 2E**). Thus, we cannot exclude the possibility that *KAI2d* do not rescue *kai2* due to low transgene expression. However, even the lowest-expressing *KAI2d* line, *PaKAI2d3* line 2, has a strong germination response to *rac*-GR24 (95% on 1 μM *rac*-GR24 vs. 12.5% on control media), indicating that its transgene expression levels are sufficient for function. Taken together with the biochemical studies of *AtKAI2* described above, the lack of seed and seedling phenotypes in SL-deficient mutants (Nelson et al., 2011; Shen et al., 2012), and the inability of carlactone to stimulate *Arabidopsis* germination (Scaffidi et al., 2013), this supports that endogenous strigolactones do not contribute significantly to *Arabidopsis* seed germination or seedling growth.

## Hypotheses and Future Directions

We hypothesize that the original function of KAI2 in land plants was KL perception, and that karrikin and strigolactone perception were derived through later adaptations of these receptors. SL-specific *KAI2* paralogs likely evolved following *KAI2* duplication in the vascular plant lineage, producing *D14*, and again within the Orobanchaceae family, producing *KAI2d* genes. Among basal vascular plants, KAI2b from *S. moellendorfii* hydrolyzes GR24^5DS^ *in vitro*. Cross-species complementation assays with *SmKAI2b* in *Arabidopsis* were not successful, so conclusions about its *in vivo* function will await reverse genetic analysis. The moss *Physcomitrella patens* has at least 10 *KAI2* paralogs; perhaps some of these genes mediate SL responses. We propose that *KAI2c* is highly conserved in parasites, even though *KAI2d* enable host-responsive germination, because KL perception serves fundamental roles in plant growth. It remains to be determined if KAI2 in non-parasitic angiosperms like *Arabidopsis* gained or improved karrikin sensitivity in addition to KL perception, or if *KAI2c* in parasites subfunctionalized to exclude non-KL ligands.

A key question now facing the field is, what is KL? At this point we can only predict that KL has a butenolide moiety, a feature shared by both classes of compounds that signal via MAX2. The identification of KL would be a significant discovery for plant biology and agriculture alike, as this may represent a new plant hormone. Of practical significance to agriculture, the *kai2* mutant demonstrates that KL promotes seed germination (or reduces seed dormancy) and seedling growth responses to light. Knockdown of *KAI2/D14L* in rice suggests a role for KL in reducing mesocotyl elongation in the dark (Kameoka and Kyozuka, 2015). In addition, KAR_1_ enhances the seedling vigor of some crops under stressful growth conditions (Jain et al., 2006; Kulkarni et al., 2006; van Staden et al., 2006), and KL may do the same. In the face of growing challenges to agriculture, KL may find uses as an agrichemical that enables more uniform germination and establishment of crops, or depletes the weed load in field seed banks. The discovery of likely receptors for KL is an important first step toward its identification. In the meantime, KARs will continue to serve as useful analogs to investigate KL biosynthesis and perception.

## Methods

Transgenic lines were described in Conn et al. (2015). Seedling assays were performed on 10 day-old seedlings grown in 8 h white light (∼36 μE):16 h dark at 21°C on 0.5× Murashige and Skoog media supplemented with 1 μM treatments or an acetone control. Hypocotyls and cotyledons were measured with ImageJ (http://rsb.info.nih.gov/ij/). qRT-PCR was performed on 10 day-old seedlings according to Stanga et al. (2013). qPCR primers span introns and are listed in Supplementary Table S1. Statistical analyses were done in JMP (SAS Institute).

## Author Contributions

CC performed the experiments, and collected and analyzed the data. CC and DN designed the research and wrote the manuscript.

## Conflict of Interest Statement

The authors declare that the research was conducted in the absence of any commercial or financial relationships that could be construed as a potential conflict of interest.
