# Supplementary material for: Evidence that KARRIKIN-INSENSITIVE2 (KAI2) Receptors may Perceive an Unknown Signal that is not Karrikin or Strigolactone
Source: Front Plant Sci. 2016 Jan 8;6:1219. doi: 10.3389/fpls.2015.01219 (PMC4705300; doi:10.3389/fpls.2015.01219)
Supplement: Supplementary file 1 [file Table_1.DOCX]

**Table S1. qPCR primer sequences**

| Gene name | Forward primer | Reverse primer |
| --- | --- | --- |
| *AtKAI2* | TGATCTCTGCTTCTCCGAGATACG | CCACGCTTTGTAGTTGCTTCGG |
| *PaKAI2c* | CGTTGGCCATTCTGTCTCGG | CGTCTTGTTCGAATCCTCCAAAG |
| *ShKAI2c* | CCAAGCTTGTCACAGTCGCCGGCTC | ATGGTCTGGAGAATGCTGAGGG |
| *ShKAI2i* | CATCATCCGCCCTGACCTCTTCC | CCTGCCAGCTGCTCCACGTCC |
| *PaKAI2d1* | CGTCGGACACTCTTTGTCTGCC | CGCCCTTCCCGATTCCAGTAAC |
| *PaKAI2d3* | TGTATCTACGTCGGCCACTCTCTG | TCCTCCTTTGTAATCAGCCGAG |
| *ShKAI2d1* | CTCTCGTCCATGGTTGCGGC | GTTTTCCTCCATGGCGGCTTGC |
| *DLK2* | GCTGCTTCTCCAAGGTATATAA | GAAATCAACCGCCCAAGCT |
| *CACS* | GGAGAAGAGAGGGCCTTGCTTACAA | TTAGCTGGGCGAGATTTCATTTCTG |
